# Supplementary material for: ERAP2 Inhibition Induces Cell-Surface Presentation by MOLT-4 Leukemia Cancer Cells of Many Novel and Potentially Antigenic Peptides
Source: Int J Mol Sci. 2022 Feb 8;23(3):1913. doi: 10.3390/ijms23031913 (PMC8836666; doi:10.3390/ijms23031913)
Supplement: Supplementary file 1 [file ijms-23-01913-s001.zip › ijms-1565750-supplementary.pdf]

# SUPPLEMENTAL DATA AND FIGURES

# ERAP2 inhibition induces cell-surface presentation by MOLT-4 leukemia cancer cells of many novel and potentially antigenic peptides

Ioannis Temponeras[1,2], George Stamatakis[3], Martina Samiotaki[3], Dimitris Georgiadis[4], Harris Pratsinis[1], George Panayotou[3] and Efstratios Stratikos[1,4,*]

[1] National Centre for Scientific Research "Demokritos", Agia Paraskevi, Attica, Greece
[2] Department of Pharmacy, University of Patras, Patra, Greece
[3] Biomedical Sciences Research Center "Alexander Fleming", Vari, Attica, Greece
[4] Department of Chemistry, National and Kapodistrian University of Athens, Greece

[*] Corresponding Author: E-mail: estratikos@chem.uoa.gr or stratos@rrp.demokritos.gr.

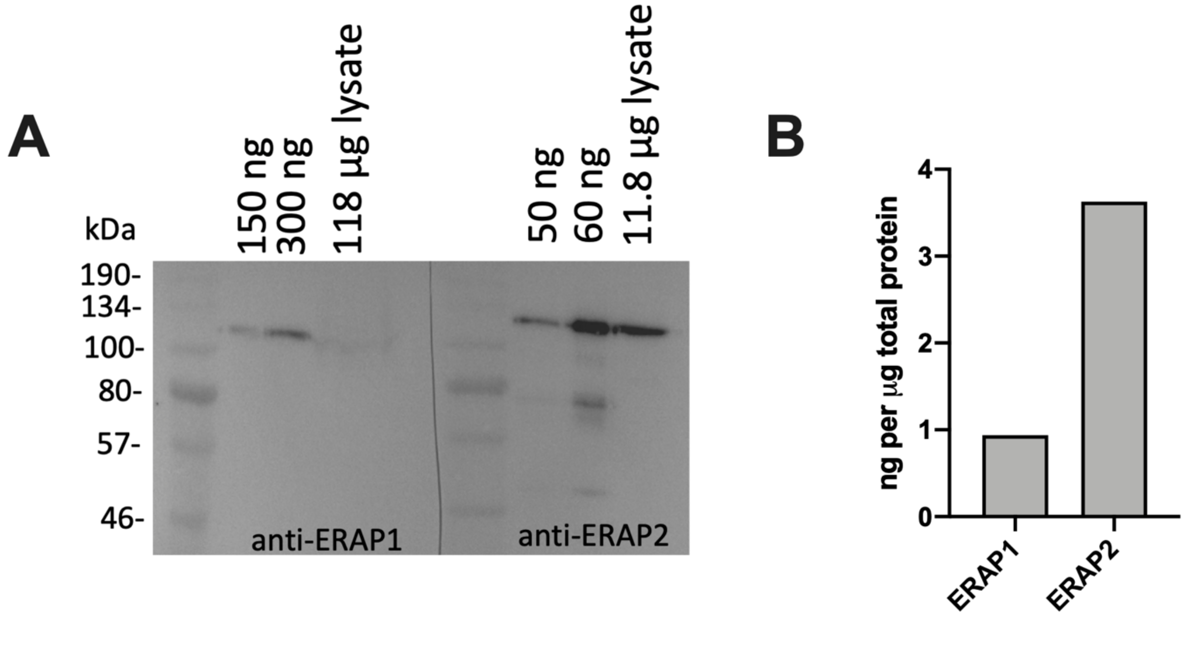


**Figure S1: Expression of ERAP1 and ERAP2 by MOLT4 cells.** Panel A, western blot using anti-ERAP1 and anti-ERAP2 antibodies from lysate from MOLT4 cells. Recombinant ERAP1 and ERAP2 were used as positive controls to evaluate the amount of proteins detected in the lysate. Panel B, quantification of ERAP1 and ERAP2 expressed in MOLT4 cells.

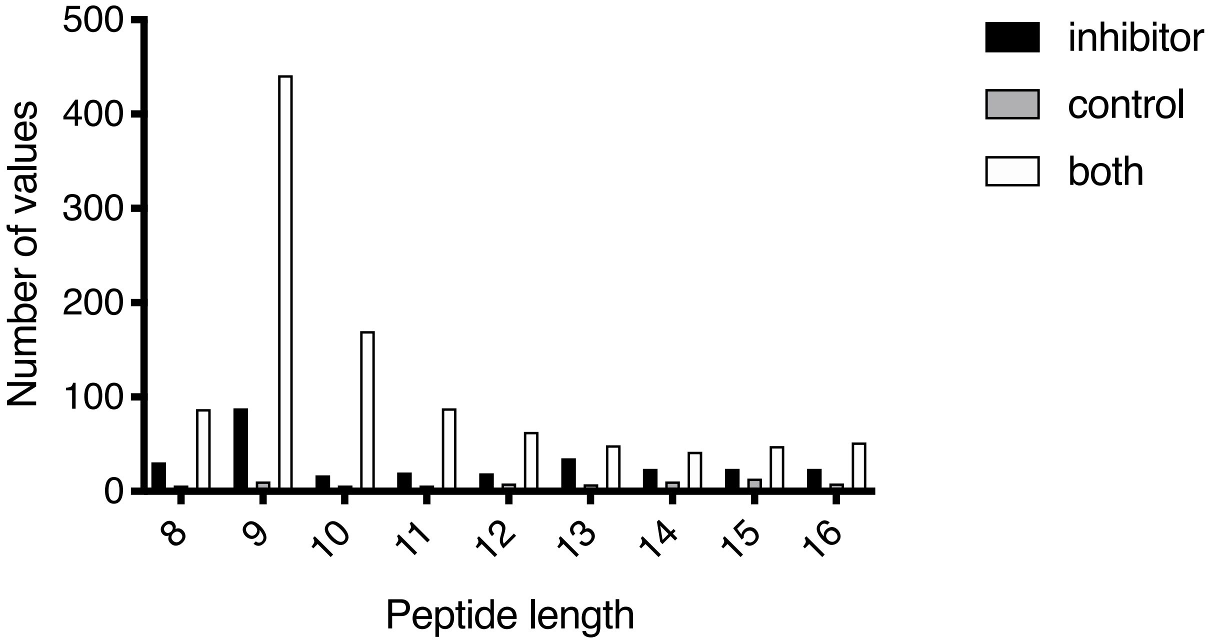


**Figure S2:** Distribution of lengths of peptide eluted from the MHC class I molecules on the surface of MOLT-4 cells. Peptides have been grouped as common in both the inhibitor and control condition, peptides unique to the control condition and peptides uniquely detected when the cells were incubated with DG011A.

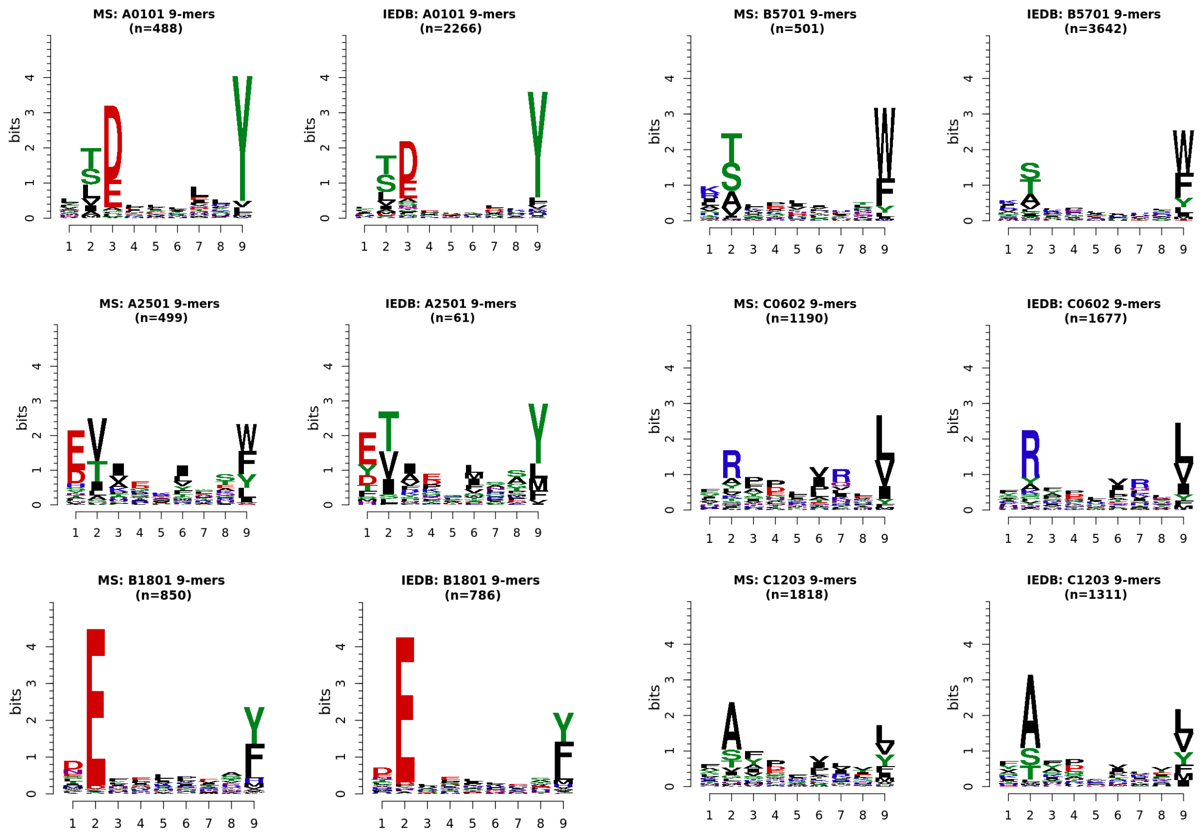


**Figure S3:** SeqLogo plots of 9mer peptides discovered to bind onto MHCI alleles expressed by MOLT4 cells. Data adapted from http://hlathena.tools/ .

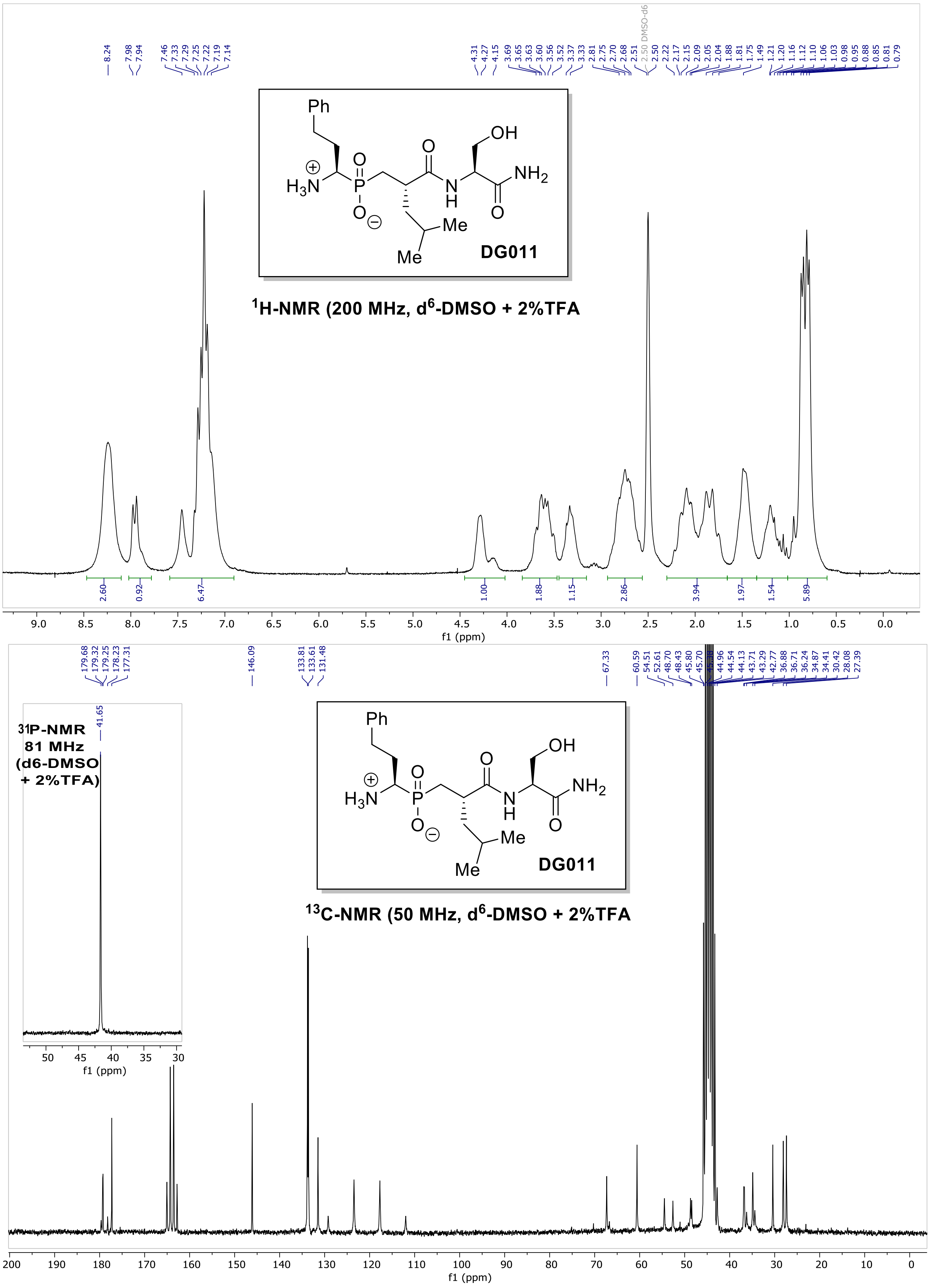


**Figure S4:** NMR spectra of DG011.

## Spectral Characterization of DG011[1]

$^{1}H$ NMR (200 MHz, DMSO-$d^6$/2% TFA) $\delta$ 0.83 (dd, J = 5.4, 11.9 Hz, 6H), 1.06 – 1.32 (m, 1H), 1.33 – 1.59 (m, 2H), 1.67 - 2.26 (m, 4H), 2.56 – 2.94 (m, 3H), 3.19 – 3.43 (m, 1H), 3.44 – 3.78 (m, 2H), 4.05 – 4.39 (m, 1H), 6.94 – 7.59 (m, 7H), 7.96 (d, J = 7.7 Hz, 1H), 8.24 (br s, 3H); $^{13}C$ NMR (50 MHz, DMSO-$d^6$/2% TFA) $\delta$ 22.4, 23.1, 25.4, 29.9, 30.3 (d, $^{1}J_{PC}$ = 92.0 Hz), 31.7, 31.9, 37.7, 37.8, 43.4, 43.7, 48.5 (d, 1JPC = 95.5 Hz), 55.6, 62.3, 126.5, 128.6, 128.8, 141.1, 172.3, 174.2, 174.3; $^{31}P$ NMR (81 MHz, DMSO-$d^6$/2% TFA) $\delta$ 41.6; HRMS (m/z): [M - H]- calcd. for $C_{19}H_{31}N_3O_5P$-, 412.2007 found, 412.2005.

[1] Kokkala, P.; Mpakali, A.; Mauvais, F.-X.; Papakyriakou, A.; Daskalaki, I.; Petropoulou, I.; Kavvalou, S.; Papathanasopoulou, M.; Agrotis, S.; Fonsou, T.-M.; van Endert, P.; Stratikos, E.; Georgiadis, D. Optimization and Structure-Activity Relationships of Phosphinic Pseudotripeptide Inhibitors of Aminopeptidases That Generate Antigenic Peptides. *J. Med. Chem.* **2016**, *59* (19), 9107–9123.

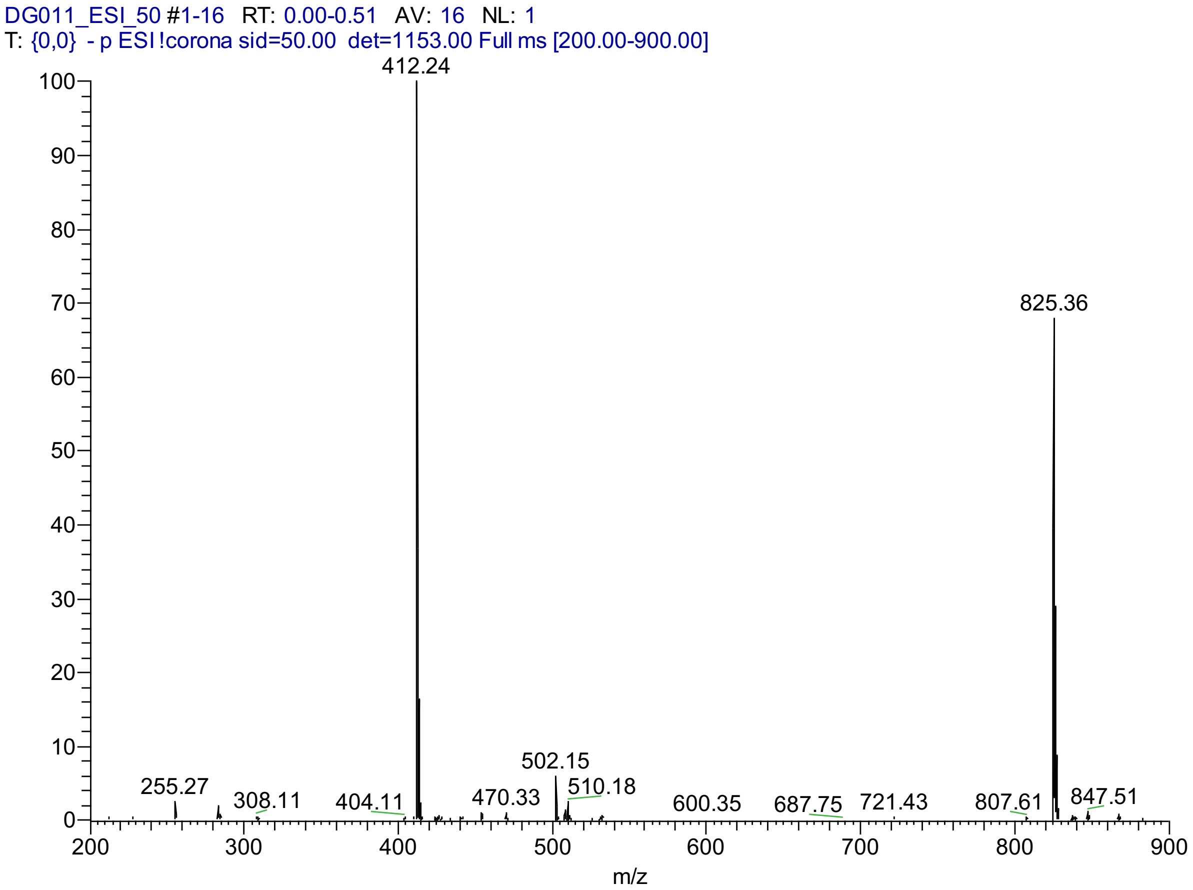


**Figure S5:** ES-MS spectrum of DG011
